# Identification of a novel intronic mutation of *MAGED2* gene in a Chinese family with antenatal Bartter syndrome

**DOI:** 10.1186/s12920-024-01797-8

**Published:** 2024-01-18

**Authors:** Xu Yan, Yueyue Hu, Xin Zhang, Xia Gao, Yang Zhao, Haiying Peng, Liu Ouyang, Changjun Zhang

**Affiliations:** 1 Reproductive Medicine Center, Renmin Hospital, Hubei University of Medicine, Shiyan, 442000 China; 2 Biomedical Engineering College, Hubei University of Medicine, Shiyan, 442000 China; 3 Obstetrics, Renmin Hospital, Hubei University of Medicine, Shiyan, 442000 China; 4 Neonatal Intensive Care, Renmin Hospital, Hubei University of Medicine, Shiyan, 442000 China; 5 Hubei Clinical Research Center for Reproductive Medicine, Shiyan, China; 6 Hubei Key Laboratory of Embryonic Stem Cell Research, Hubei University of Medicine, Shiyan, China

**Keywords:** Antenatal Bartter syndrome, Whole exome sequencing, *MAGED2*, Intronic variant

## Abstract

**Background:**

Antenatal Bartter syndrome is a life-threatening disease caused by a mutation in the *MAGED2* gene located on chromosome Xp11. It is characterized by severe polyhydramnios and extreme prematurity. While most reported mutations are located in the exon region, variations in the intron region are rarely reported.

**Methods:**

In our study, we employed whole exome sequencing and Sanger sequencing to genotype members of this family. Additionally, a minigene assay was conducted to evaluate the impact of genetic variants on splicing.

**Results:**

Our findings reveal a novel intronic variant (NM_177433.3:c.1271 + 4_1271 + 7delAGTA) in intron 10 of the *MAGED2* gene. Further analysis using the minigene assay demonstrated that this variant activated an intronic cryptic splice site, resulting in a 96 bp insertion in mature mRNA.

**Conclusions:**

Our results indicate that the novel intronic variant (c.1271 + 4_1271 + 7delAGTA) in intron 10 of the *MAGED2* gene is pathogenic. This expands the mutation spectrum of *MAGED2* and highlights the significance of intronic sequence analysis.

**Supplementary Information:**

The online version contains supplementary material available at 10.1186/s12920-024-01797-8.

## Introduction

Bartter syndrome is a rare genetic disorder caused by mutations in genes that encode various components of the renal tubular transport system, such as K^+^ channel (*KCNJ1* gene), Cl^–^ channel (*CLCNKA* and *CLCNKB* genes), their cotransporters (*SLC12A1* gene), subunits of these channels (*BSND* gene), or regulators of the expression of certain transport channels (*MAGED2* gene). Among these genes, *MAGED2* mutations are associated with the transient form of Bartter syndrome, which is usually called Antenatal Bartter syndrome caused by mutations in the *MAGED2* gene located on chromosome Xp11 [1, 2]. It is inherited in an X-linked recessive manner and can potentially be a life-threatening condition. This syndrome is characterized by transient renal salt wasting and fetal polyuria, leading to severe polyhydramnios, premature delivery, and increased perinatal mortality. However, survivors of this condition typically experience spontaneous recovery [3].

The *MAGED2* gene encodes the protein melanoma-associated antigen D2 (MAGE-D2), which is expressed in tubules outside the thick ascending limb of the loop of Henle in both the fetal and adult kidney. MAGE-D2 plays a crucial role in regulating the expression of two important transporters, NKCC2 and NCC [1, 4]. These transporters are involved in the urinary concentration and volume regulation processes. Disruptions in the expression or activity of NKCC2 and NCC due to *MAGED2* mutations can lead to the characteristic symptoms observed in Antenatal Bartter syndrome [5].

Hemizygous mutations in the coding region or canonical splice sites of the *MAGED2* gene have recently been associated with severe polyhydramnios and prematurity in male patients [1, 6]. Emerging evidence suggests that intronic mutations can contribute to the development of human diseases by impacting splicing processes [7–9]. Whole exome sequencing (WES) has the capacity to sequence the complete coding region of all known genes, including adjacent intronic regions, allowing for the identification of additional intronic variants with adequate read depth [8, 10].

In this study, we present the cases of two pregnancies in a Chinese family, involving both male and female fetuses, that were complicated by severe polyhydramnios. The condition was diagnosed at 20 weeks of gestation, and both fetuses were found to carry a novel intronic variant (c.1271 + 4_1271 + 7delAGTA) in the *MAGED2* gene. Through minigene assay analysis, we discovered that this variant led to the activation of an intronic cryptic splice site and resulted in a 96 bp insertion in the mature mRNA.

## Materials and methods

### Study subjects and medical history

**Fig. 1:**
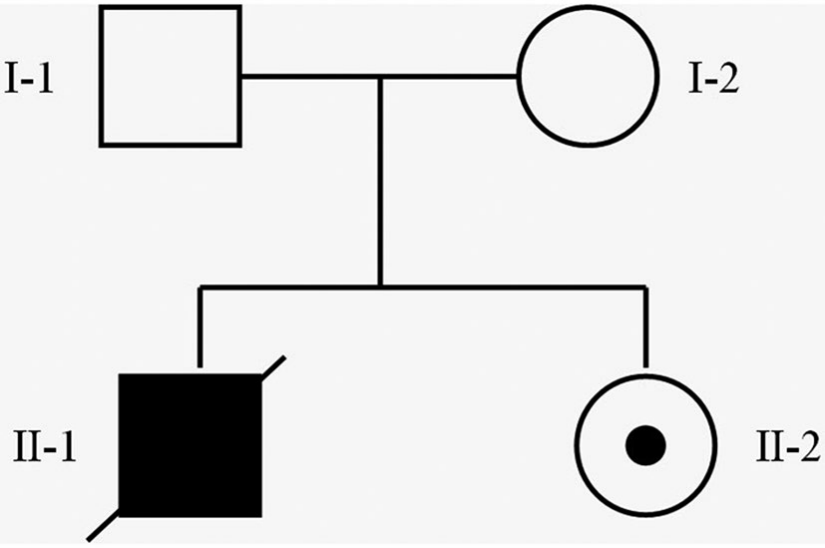
The pedigrees of the family with transient antenatal Bartter’s syndrome

The first pregnancy of this family was complicated by severe polyhydramnios that was diagnosed at 20 weeks of gestation without treatment of indomethacin. Premature rupture of the membranes in this case happened at 25 weeks of gestational age and the mother was admitted to hospital treated with ritodrine and magnesium sulfate to inhibit contractions before birth. A male infant was delivered vaginally at the 32 week of pregnancy with the birth weight of 2.7 kg and birth length was 50.8 cm. His 1- and 5-minute Apgar scores were 4 and 8. Due to neonatal asphyxia and poor response after CPR, the infant was admitted to neonatal department 30 min later. He died 8 h later with clinical diagnosis of metabolic acidosis, neonatal asphyxia, respiratory distress and right ventricular cardiomyopathy. The second pregnancy was also complicated by severe polyhydramnios that was diagnosed at 20 weeks of gestation and started taking enteric-coated indomethacin tablets at 22 weeks and 2 days of gestation. The initial dosage was 50 mg, with a reduced dosage of 25 mg on the first day. Starting from the second day, the dosage was adjusted to 25 mg four times a day. At 25 weeks and 2 days of gestation, the dosage was further adjusted to 50 mg three times a day. Indomethacin was discontinued at 29 weeks and 3 days of gestation. At 27 weeks and 5 days of gestation, amniocentesis was performed due to excessive amniotic fluid, with a total volume of 1500 ml extracted. Post-procedure, the patient received tocolytic therapy with atosiban and was discharged four days later. At 38 weeks and 3 days of gestation, the patient delivered a female infant via cesarean section. The infant had an Apgar score of 9 at 1 min and 10 at 5 min after birth. The infant stayed at a confinement center for one month after birth, with daily measurements of intake and output. The approximate daily intake was around 600 ml, while the output was approximately 400 ml. During this period, electrolyte levels, renal function, and blood gas analysis were rechecked and showed no significant issues (Fig. 1).

### Genetic analysis

Genetic analysis of amniotic fluid cells was performed during the first pregnancy at 20 weeks gestation and during the second pregnancy at 21 weeks gestation. Both pregnancies were subject to to the same genetic analysis procedures (karyotype analysis and chromosomal copy number variation (CNV) analysis). For the second pregnancy, additional trio-whole-exome was performed on the amniotic fluid cells. The first child’s peripheral blood underwent individual exome sequencing. The amniotic fluid cells were cultured and subjected to G-banding using standard procedures [11], with a resolution of 320–400 bands. Chromosomal copy number variations analysis was performed using low-depth whole-genome sequencing, utilizing the BGISeq-500 sequencing platform. The sequencing parameters were single-end sequencing, with a read length of 35 bp and a minimum of 18 million reads [12]. Whole-exome sequencing was performed using the SureSelect Human All Exon V6 capture chip, with paired-end sequencing and a read length of 100 bp [13]. The average sequencing depth was 180X.

Sanger sequencing was needed to conform the likely pathogenic mutation sites in the proband and his parents.

### In vitro mini-gene splicing assay

The *MAGED2* c.1271 + 4_1271 + 7delAGTA variant is located within intron 10. To investigate its impact, we amplified the wild-type fragments (exon10 (63 bp) - intron10 (1100 bp) - exon11 (115 bp)) and mutant fragments (exon10 - intron10 (c.1271 + 4_1271 + 7delAGTA) - exon11) using PCR. These fragments were then inserted into the pcDNA3.1 vector. Human cell lines (HeLa and 293T cells) were separately transfected with the wild-type and mutant constructs. After 48 h of culturing, total RNA was extracted, and RT-PCR was performed to analyze the splicing patterns of the wild-type and mutant variants.

## Results

### Karyotype and chromosomal CNV analysis

The karyotype analysis of the fetal cells obtained from the amniotic fluid revealed a normal karyotype of 46, XN, without any structural abnormalities at the 320–400 band level in both pregnancies. Furthermore, no chromosome aneuploidy, microdeletions, or microduplications larger than 100 kb with known pathogenicity were detected in either of the two pregnancies.

In the first pregnancy, two microduplications of uncertain significance were identified. One was located on the short arm of chromosome 9 (21,000,521 − 21,109,399), and the other was found on the long arm of chromosome 15 (30,364,215 − 31,096,810). It is important to note that there have been no reported diseases associated with these specific regions. Additionally, these regions do not contain any pathogenic genes listed in OMIM and do not have any pathogenic or benign records in databases such as ISCA, Decipher, ClinVar, and others. Interestingly, the microduplication of uncertain significance on the short arm of chromosome 9 (21,000,521 − 21,109,399) was also observed in the second pregnancy. As with the first pregnancy, there is no known disease associated with this region.

### Whole exome sequencing results

In the whole exome sequencing analysis, the mean coverage depth was 180x, with 95% of the target bases covered at least 20x. Notably, the entire coding region of the MAGED2 gene was completely covered, facilitating the detection of the identified splice variant. The sequencing reads were aligned to the human genome (hg19) using the BWA tool. Optional steps, such as duplicate marking and BAM file sorting, were performed using the GATK tool. To enhance the quality of the sequenced bases, the Broad Institute’s algorithms were applied, specifically the Base Quality Score Recalibration (BQSR) process. The resulting BAM files were then subjected to variant calling using GATK HaplotypeCaller. Using the generated VCF file and a set of phenotypes (HP:0001561:Polyhydramnios, HP:0001622: Premature birth), the Exomiser tool identified a potential disease-causing variant (c.1271 + 4_1271 + 7delAGTA) that could disrupt the coding sequence of *MAGED2*. This variant is predicted to be deleterious and is associated with antenatal Bartter syndrome, a condition characterized by fetal polyuria, polyhydramnios, and prematurity. Interestingly, this mutation was found in both the firstborn male child and the second-born female child, while it was not detected in their parents.

In ensuring the specificity of the findings, we meticulously examined other significant genes associated with Bartter syndrome, including *SLC12A1*, *KCNJ1*, *CLCNKB*, *CLCNKA*, and *BSND*. Through the whole exome sequencing approach, we effectively ruled out the presence of disease-causing variants in these genes, consolidating the likelihood of the pathogenic role of the novel splice variant identified in the MAGED2 gene.

### Results of the mini-gene splicing assay

**Fig. 2:**
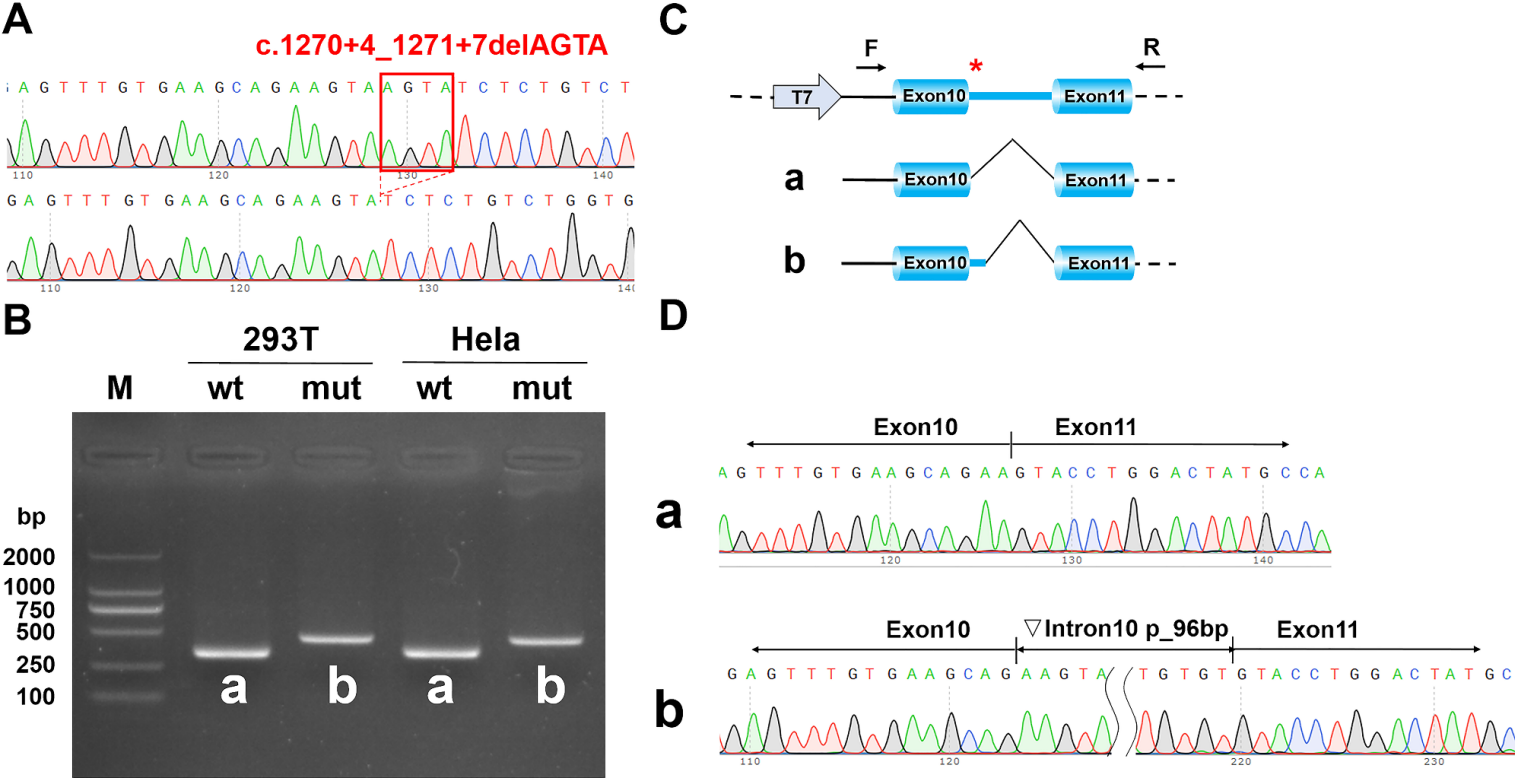
Results of the mini-gene splicing assay. (**A**) sanger sequencing of mini-gene construction, the upper panel is the sequence of wild type, the lower panel is the sequence of mutant type (c. 1271 + 4_1271 + 7delAGTA); (**B**) Gel electrophoresis of RT-PCR products; (**C**) schematic diagram of mini-gene construction and the mutant type splicing; (**D**) The mutant mini-gene caused a splicing abnormality, resulting in the retention of the 96 bp in intron 10

The mini-gene splicing products were analyzed using PCR amplification and agarose gel electrophoresis. The electrophoresis results of wild-type and c.1271 + 4_1271 + 7delAGTA transfections revealed that the mutant band exhibited a larger size compared to the wild-type band, resulting in a slower migration. The amplicons demonstrated that the cDNA fragment obtained from the c.1271 + 4_1271 + 7delAGTA plasmid contained an additional 96 bp in intron 11 to exon 11, which was confirmed by Sanger sequencing (Fig. 2). This mutation would cause the codon 425 (TAC) to change into a stop codon (TAA).

## Discussion

Children with Bartter syndrome typically present with symptoms such as polyhydramnios, premature delivery, polyuria, polydipsia, signs of hypovolemia, failure to thrive, and poor growth [14]. Bartter syndrome is classified into five major genotypes caused by mutations in different genes, namely *SLC12A1*, *KCNJ1*, CLCNKB, *CLCNKA*, *BSND*, and *MAGED2* [15, 16]. The antenatal form, specifically type 5, of Bartter syndrome caused by *MAGED2* gene mutation is a life-threatening condition. It manifests with significant fetal polyuria leading to polyhydramnios between 24 and 30 weeks of gestation, resulting in premature delivery. The onset of polyhydramnios and labor in antenatal Bartter syndrome occurs earlier compared to other forms of Bartter syndrome, and the symptoms typically resolve spontaneously after childbirth [2].

**Fig. 3:**
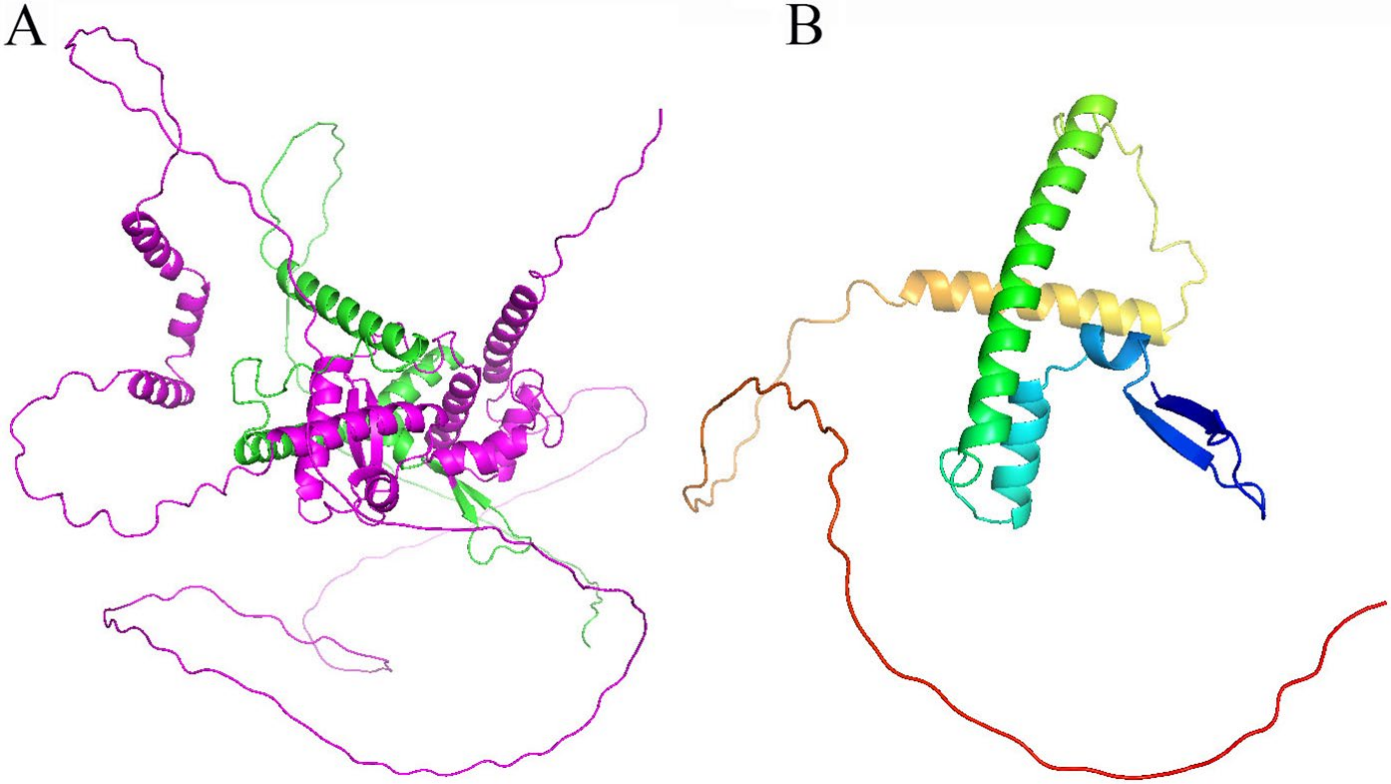
The predicted 3D structure of Melanoma-associated antigen D2 (MAGE-D2) using AlphaFold is shown. (**A**) The structure depicts the full-length of MAGE-D2. (**B**) The structure specifically highlights the C-terminal region (amino acid residues 425–606) of MAGE-D2

In our case, the first fetus was born as a male baby with earlier clinical features of polyhydramnios, which is consistent with the x-linked recessive inheritance and antenatal Bartter-like phenotype. By whole exome sequencing, we identified a novel intronic variant (c.1271 + 4_1271 + 7delAGTA) in the *MAGED2* gene. Furthermore, the mini-gene splicing assay revealed that this mutation would cause the codon 425 (TAC) to change into a stop codon (TAA) and the translation would stop at Tyr425. The structure of the MAGE-D2 protein has not been resolved by experimental methods. However, using AlphaFold predictions, we obtained an approximate model of its overall structure. It is predominantly composed of 13 α-helices and 4 β-strands. The predicted confidence score for the N-terminal residues is around 70, while for the C-terminal residues, it is around 90 (Fig. 3). The missing C-terminal region (amino acid residues 425–606) consists of 4 α-helices and 2 β-strands, with a confidence score of approximately 90 [17]. We speculate that this model represents the interaction region between the MAGE-D2 protein and Hsp40. Its deletion prevents MAGE-D2 from binding to Hsp40, thereby impairing the protection of NKCC2 and NCC from endoplasmic reticulum–associated degradation mediated by Hsp40 [1].

After the first pregnancy, the genetic counselor informed the couple that this variant is a de novo mutation and the recurrence risk is very low, so a regular prenatal check-up for the next pregnancy is sufficient. Unfortunately, despite being a female fetus in the second pregnancy, excessive amniotic fluid occurred again at 20 weeks of gestation. Genetic testing is performed at the 21st week of pregnancy, which is very close in timing to the genetic testing conducted at 20 weeks during the first pregnancy. The first genetic testing included karyotype analysis and chromosomal CNV analysis followed by whole exome sequencing. In this round of genetic testing, trio-whole-exome sequencing is carried out in addition to karyotype analysis and chromosomal CNV analysis. This has significantly shortened the genetic testing timeline, providing better support for clinical decision-making. Through whole-exome sequencing of amniotic fluid cells, the same pathogenic variant (c.1271 + 4_1271 + 7delAGTA) in the *MAGED2* gene was identified in the second child. Based on this, we have reason to believe that the mother is a carrier of germline mosaicism although the addition experiments confirming the gonadal mosaicism were not performed due to the inconvenience of obtaining samples, and X-chromosome skewed inactivation has occurred in the second child [18].

While whole exome sequencing offers a comprehensive view of the coding regions of the genome and the potential to identify novel variants, it also presents challenges such as increased data complexity and the need for stringent bioinformatics pipelines. In contrast, targeted panels provide a focused analysis, enhancing the detection of variants in specific genes of interest, yet they may miss novel variants in genes that are not included in the panel. A thorough understanding of these considerations can aid in the selection of the most suitable approach for precise and effective Prenatal Diagnosis (PND) in Bartter syndrome.

## Conclusion

Although various types of mutations associated with transient antenatal Bartter syndrome, such as frameshift, splice-site mutations, in-frame deletions, nonsense, and missense mutations, have been identified, mutations in the intron region have been rarely reported [1, 4, 19]. In this report, we present a case of antenatal Bartter syndrome with a novel pathogenic deletion in the intron of the *MAGED2* gene and we report here, for the first time, a female fetus carrying a pathogenic heterozygous variant in the *MAGED2* gene, which can also result in antenatal Bartter syndrome.

Considering that polyhydramnios and postnatal polyuria phenotypes are caused by multiple genes and that pathogenic (or likely pathogenic) variations reported thus far mainly consist of single nucleotide variants and small insertion-deletions [20–24], whole-exome sequencing is recommended for pregnant woman and preterm infants. Moreover, the cost of next-generation sequencing is now acceptable, making it a feasible option. Genetic testing results are crucial for informing future reproductive decisions and minimizing the risk of recurrence.

Based on our case, it is important to emphasize two points during genetic counseling. Firstly, novel mutations may be caused by germline mosaicism, which could pose a certain risk of recurrence. Secondly, if a female carries an X-linked recessive pathogenic variant, it is crucial to consider X-chromosome skewed inactivation [18], as it may lead to the manifestation of the corresponding disease in her offspring.

### Electronic supplementary material

Below is the link to the electronic supplementary material.

**Supplementary Material 1: Supplementary Figure 1.** Gel electrophoresis of RT-PCR products in Figure 2 with full length membranes. The agarose gel electrophoresis lanes from left to right are as follows: Lane 1 is the marker (molecular weights ranging from small to large: 100bp, 250bp, 500bp, 750bp, 1000bp, 2000bp); Lanes 2 and 3 represent the spliced bands of pcDNA3.1-MAGED2-wt/mut in 293T cells; Lanes 4 and 5 represent the spliced bands of pcDNA3.1-MAGED2-wt/mut in HeLa cells

## Data Availability

All the data generated or analyzed during this study are available from the corresponding author by reasonable request. The identified variant in this research is accessible on the ClinVar repository under accession number “SUB14147953” for the *MAGED2* gene mutation.
