# Supplementary material for: Identification of a novel intronic mutation of MAGED2 gene in a Chinese family with antenatal Bartter syndrome
Source: BMC Med Genomics. 2024 Jan 18;17:23. doi: 10.1186/s12920-024-01797-8 (PMC10795325; doi:10.1186/s12920-024-01797-8)
Supplement: Supplementary file 1 — Supplementary Material 1: Supplementary Figure 1. Gel electrophoresis of RT-PCR products in Figure 2 with full length membranes. The agarose gel electrophoresis lanes from left to right are as follows: Lane 1 is the marker (molecular weights ranging from small to large: 100bp, 250bp, 500bp, 750bp, 1000bp, 2000bp); Lanes 2 and 3 represent the spliced bands of pcDNA3.1-MAGED2-wt/mut in 293T cells; Lanes 4 and 5 represent the spliced bands of pcDNA3.1-MAGED2-wt/mut in HeLa cells [file 12920_2024_1797_MOESM1_ESM.docx]

Supplementary Material


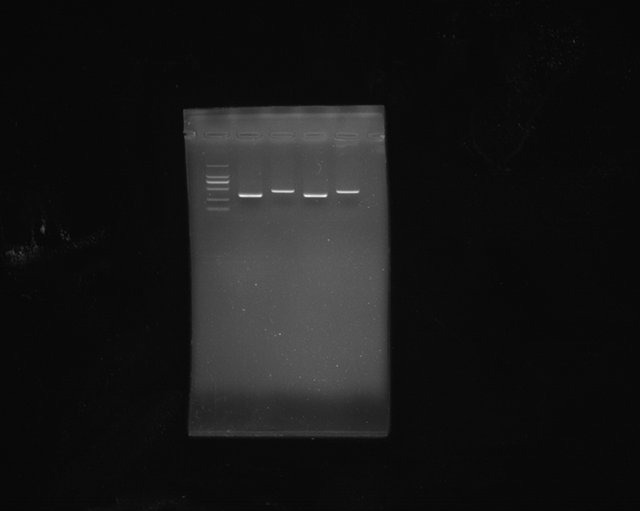


Supplementary Figure 1. Gel electrophoresis of RT-PCR products in Figure 2 with full length membranes. The agarose gel electrophoresis lanes from left to right are as follows: Lane 1 is the marker (molecular weights ranging from small to large: 100bp, 250bp, 500bp, 750bp, 1000bp, 2000bp)； Lanes 2 and 3 represent the spliced bands of pcDNA3.1-MAGED2-wt/mut in 293T cells；Lanes 4 and 5 represent the spliced bands of pcDNA3.1-MAGED2-wt/mut in HeLa cells.
